# *In vitro* and *in vivo* antitumor effect of gefitinib nanoparticles on human lung cancer

**DOI:** 10.1080/10717544.2017.1384862

**Published:** 2017-09-29

**Authors:** Xiao Ling Ni, Long Xia Chen, Heng Zhang, Bo Yang, Shan Xu, Min Wu, Jing Liu, Ling Lin Yang, Yue Chen, Shao Zhi Fu, Jing Bo Wu

**Affiliations:** a Department of Oncology, the Affiliated hospital of Southwest Medical University, Luzhou, China;; b Department of Nuclear Medicine, the Affiliated hospital of Southwest Medical University, Luzhou, China

**Keywords:** Gefitinib, PCEC, nanoparticles, lung cancer

## Abstract

Gefitinib (GEF) is the first epidermal growth factor receptor (EGFR)-targeting agent launched as an anticancer drug. It is an accepted opinion that modifying GEF strong hydrophobicity and poor bioavailability would not only enhance its antitumor effects, but also reduce its side effects. In this study, GEF-loadedpoly(ε-caprolactone)-poly(ethyleneglycol)-poly(ε-caprolactone) (PCEC) -bearing nanoparticles (GEF-NPs) were prepared by a solid dispersion method and characterized. The particle sizes increased with the increase in GEF/PCEC mass ratio in feed. GEF-NPs (10%) were mono-dispersed, smaller than 24 nm, zeta potential was approximately −18 mV, percentage encapsulation and loading, were more than 9% and 92%, respectively, and drug was slowly released but without a biphasic pattern. Microscopy studies of the optimized formulation confirmed that the prepared nanoparticles are spherical in nature. Cytotoxicity results indicated that cell growth inhibition induced by free GEF and GEF-NPs were dose and time dependent. Compared with free GEF, GEF-NPs enhanced antitumor effects, reduced side effects and significantly prolonged survival time *in vivo*. CD31, ki-67 and EGFR expression were significantly lower in the GEF-NPs group compared with other groups (*p<* .05). These findings demonstrated that GEF-NPs have the potential to attain superior outcomes and to overcome complications such as organs toxicity, therapeutic resistance and disease relapse.

## 1. Introduction

Lung cancer is the foremost cause of cancer-related deaths worldwide (Herbst et al., 2008). Nonsmall-cell lung cancer (NSCLC) is the most common lung cancer pathological type and its treatment includes surgical resection, chemoradiotherapy, targeted therapy and comprehensive treatment. Targeted therapies, which now belong to precision oncology, are revolutionizing the treatment of advanced NSCLC. Because of tumor heterogeneity, the same tumor type shows difference in gene repression, leading to different response to treatments, tumor invasion and metastasis ability. The discovery of key oncogenic events mainly in NSCLC, like epidermal growth factor receptor (EGFR) mutations or anaplastic lymphoma kinase rearrangements, have changed dramatically the therapeutic strategy with the introduction of tyrosine kinase inhibitors (TKIs) such as gefitinib (GEF), which regulates tumor progression and metastatic spread attitude with an improvement of symptoms control and patients’ quality of life compared with traditional cytotoxic agents (Maemondo et al., 2010).

GEF is a selective EGFR TKI and usually used in the treatment of NSCLC, since it exerts an antineoplastic effect by blocking EGFR signaling (Rahman et al., 2014). EGFR, which is expressed on the cell surface of normal cells and overexpressed in many cancer cells and associated with cancer cell proliferation, metastasis and survival (Takeda & Nakagawa, 2015). GEF is in the form of a white powder, sparingly soluble at pH 1; its solubility rapidly decreases in the upper gastric range, especially at pH 4–6 and is practically insoluble above pH 7 (Bergman et al., 2007). Its poor solubility in gastric fluid weakens the action onset, bioavailability and therapeutic activity. GEF log *p* value is 3.2, indicating that it is highly lipophilic (Rahman et al., 2014). GEF oral bioavailability is less than 44% in human (Wilson et al., 2009). In addition, because of the widely distribution of EGFR in normal tissues, treatment with GEF is accompanied with a number of side effects including skin rash (Pastore et al., 2008), diarrhea, interstitial lung disease, left ventricular dysfunction (Jacob et al., 2016). Therefore, the development of new GEF delivery systems to increase bioavailability and decrease distribution in normal tissues is highly demanded.

The nanoparticulate drug delivery system brings hope to solve the aforementioned problems. Encapsulation of active compounds into polymeric carriers represents a very promising way of increasing drug bioavailability, preventing drug degradation, reducing drug toxic effects, controlling drug release and achieving specific targeting. In recent years, many new GEF nano prescriptions brought out in all over the world. Lee et al. synthetized GEF–cyclodextrin inclusion complexes and the solubility rate of the drug was significantly increased (Lee et al., 2009). Trummer et al. developed a nanoliposomal GEF formulation in 2012 (Trummer et al., 2012). Colloidal gold nanoparticle also can be successfully employed for conjugating GEF, as shown by Lam et al. (Lam et al., 2014). GEF-loaded folate-decorated bovine serum albumin-conjugated carboxymethyl-β-cyclodextrin nanoparticles were produced by Shi et al. and resulted in enhanced drug delivery and attenuated autophagy in folate receptor-positive cancer cells (Shi, 2014). Zhao et al. found that codelivery of GEF and chloroquine by chitosan nanoparticles could overcome GEF acquired resistance (Zhao et al., 2015). Although GEF nano-controlled released system with hydroxypropyl β-cyclodextrin, chitosan, colloidal gold, liposomal and many other compounds were already used for formulations design, all of them lacked the *in vivo* evaluation of GEF nano-drugs antitumor effect.

Compared with these mentioned nano-formulations, polymeric nanoparticles have many advantages in drug delivery systems (De et al., 2010): firstly, they have strong antidilution ability and they are stable in circulatory system, next, their shell nanosize and hydrophilic properties can prevent reticuloendothelial system identification during *in vivo* absorption, thus lengthening the time in the blood circulating system. Lastly, due to the enhanced permeability and retention (EPR), nanoparticles small size (10 ∼ 200 nm) is beneficial to the tumor tissue retention and accumulation, making them ideal for passive targeting of tumor tissue. Therefore, polymeric nanoparticles represent a promising drug delivery system for hydrophobic antitumor drugs (Mora-Huertas et al., 2010).

Poly(ε-caprolactone)-poly(ethyleneglycol)-poly(ε-caprolactone) (PCEC) is a chemical synthesized tri-block copolymer, which belongs to the class of hydrolytically degradable polymers. Because of its biodegradability, biocompatibility, amphiphilic and appropriate mechanical properties, it is widely used as carrier of many antitumor drugs (Yadav et al., 2010; Yang et al., 2017). The purpose of our study was to prepare GEF-loaded PCEC nanoparticles (GEF-NPs), evaluate its anti-tumor effect and its mechanism on human lung carcinoma *in vitro* and *in vivo*, especially considering the assessment of normal tissue toxicity.

## 2. Material and methods

### 2.1. Materials

Poly (ethylene glycol) (PEG, Alfa Aesar), ε-caprolactone (ε-CL) (Alfa Aesar, Reston, VA), stannous octoate (Sn(Oct)_2_), RPMI-1640 and 3-(4,5-dimethylthiazol-2-yl)-2,5-diphenyltetrazolium bromide (MTT) were purchased from Sigma (St. Louis, MO), GEF was purchased from Meilun Co., Ltd (Dalian, China). Dimethylsulfoxide (DMSO), anhydrous ethanol, methanol (HPLC grade) and isopropyl alcohol were purchased from KeLong Co., Ltd (Chengdu, China). IL-6 ELISA Kit and TGF-β1 ELISA Kit were purchased from ChengLin Biological Technology (Beijing, China). Hydroxyproline assay kit was purchased from Nanjing Institute of Biological Engineering (Nanjing, China). Ki-67, EGFR and CD31 polyclonal antibody were purchased from Bioworld Technology (Nanjing, China).

### 2.2. Cell lines and animals

Human lung carcinoma cells (A549) were obtained from the Experimental Medicine Center, at the affiliated hospital of Southwest Medical University (Luzhou, China). A549 were incubated in RPMI medium (1640, Gibco, Grand Island, NY) supplemented with 10% heated-inactivated fetal bovine serum (FBS, Gibco, Grand Island, NY), 1% penicillin-streptomycin and maintained at 37 °C, under 5% CO_2_ in a humidified incubator.

BALB/c athymic nude mice (female, 3–4 weeks old, weighing 14–18 g) were purchased from Chongqing TengXin Biotechnology Co., Ltd (Chongqing, China). Guidelines of the China Council on Animal Care were followed, such as ad libitum access to food and water, controlled room temperature (20–22 °C) and relative humidity of 50–60%, 12-h light/dark cycle. All the procedures were ethically and scientifically approved by the Institutional Animal Care and Treatment Committee of Southwest Medical University (Luzhou, China).

### 2.3. GEF-NPs preparation

PCEC (*M*_w_=3700) was synthesized by ring-opening polymerization, according to a previous report (Jia et al., 2008). Briefly, a calculated PCEC and GEF amount was completely co-dissolved in anhydrous ethanol. The mixed solution was evaporated in a rotator evaporator (60 °C, 110 ± 5 rpm) to remove the organic solvent, and the resulting harvested film was washed using preheated (60 °C) deionized water and filtered by 220-nm water filters to obtain a clarified solution for further characterization. The filtrate was freeze dried to produce the GEF-NPs powder.

### 2.4. Nanoparticles physicochemical characterization

GEF-NPs surface morphology was observed using atomic force microscope (AFM, Dimension Icon, Bruker Co., Germany). GEF-NPs particle size was evaluated three times by dynamic light scattering (DLS, NanoBrook90 plus Zeta, Brookhaven, NY) at 25 °C.

Drug loading (DL) and encapsulation efficiency (EE) were determined by HPLC. The optimal chromatographic conditions were conformed as follows: Agilent 1260 HPLC (Milford, MA) system equipped with four pumps, an auto-sampler and DAD. The analytical column was a reversed-phase C18 alkyl silane column (150 mm × 4.6 mm, 5 μm, Agilent Santa Clara, CA) at 30 °C. The mobile phase consisting of methanol and 0.1 M potassium dihydrogen phosphate at the ratio of 40:60 (v/v) and at a flow rate of 1.0 mL/min (the pH was adjusted to pH 3.2 with phosphoric acid solution) was degassed 30 min before use. The detection wavelength was set at 254 nm and injection volume was 20 μL. Because of the use of high concentration of phosphate, it is necessary to operate as follows to allow the block of HPLC system: the composition of the mobile phase at time zero was 95% potassium dihydrogen phosphate (0.1 M, pH 3.2) and 5% methanol, the percentage of methanol was gradually increased to 40% within 40 min. DL and EE were calculated in accordance with the following equations:

$$\text{DL\%}=\frac{\text{Drug}}{\text{(Polymer }+\text{ Drug)}}\;\times \text{ 100\% }$$

$$\text{EE\% }=\;\frac{\text{Actual DL}}{\text{Theoretical DL}}\;\times \text{ 100\% }$$

### 2.5. *In vitro* release behavior

To explore GEF *in vitro* release behavior from GEF-NPs, 1 mL (1 mg/mL) free GEF dissolved in dehydrated alcohol or 1 mL GEF-NPs (freeze-dried powder reconstituted with distilled water, equal to 1 mg of GEF) were placed in dialysis bags (molecular weight cutoff, 3.5 kDa). The dialysis bags were incubated in 30 mL phosphate buffer (pH 7.4) containing Tween80 (0.5%, w/w) at 37 °C under gentle shaking (100 rpm). Incubation medium was replaced by the same volume of fresh pre-heated medium at specific time points (2 h, 4 h, 6 h, 8 h, 10 h, 12 h, 24 h, 48 h, 72 h, 96 h and 120 h). The released drug was collected by centrifuging at 12,000 rpm for 15 min at 4 °C. The supernatant was collected and stored at −20 °C for HPLC analysis, and this measurement was repeated at least three times.

### 2.6. MTT assay

Briefly, A549 cells were plated in 96-well plates at a density of 5 × 10^4^ cells per well in 150 μL RPMI medium and incubated for 24 h. Cells were then exposed to free GEF, blank PCEC (PCEC without GEF) and GEF-NPs at different concentrations for 24 h or 48 h, and the results were measured using MTT assay (Denizot & Lang, 1986). Absorbance (A) of each well was recorded at 490 nm by a microplate reader (iMark, Hercules, CA). Cell viability was calculated by the following equation: cell viability (%) =*A*_treated_/*A*_control_×100%.

### 2.7. *In vitro* cellular uptake

To evaluate PCEC nanoparticles uptake by A549 cells, fluorescent probe coumarin-6 (C6) was encapsulated in PCEC nanoparticles. Preparation of C6-labeled blank PCEC nanoparticles was performed as follows: C6 dichloromethane solution was dropped into PCEC absolute ethyl alcohol solution, with a molar ratio PCEC:C6 controlled at 1:4. Then, the mixed solution was evaporated in a rotator evaporator as described in the Materials 2.3. A549 cells were seeded in a six-well plate at approximately 1 × 10^5^ cells mL^−1^ per well in 2 mL RPMI medium and incubated for 24 h. Next, 10 μL C6-labeled PCEC nanoparticles, PBS (pH 7.4), were added to the six-well plate and the cells were further incubated for 2 h. Cells were observed by fluorescence microscopy after washing three times with PBS (pH 7.4).

### 2.8. GEF-NPs *in vivo* antitumor effect

Nude mice received a subcutaneous injection of A549 cell suspension 100 μL (containing 1 × 10^6^ cells) into the right flank. When the mean tumor volume reached approximately 100–200 mm^3^, tumor-bearing mice were randomly divided into the following four treatment groups (*n* = 12 mice per group): (1) normal saline (NS), (2) blank PCEC nanoparticles (PCEC), (3) free GEF (20 mg/kg), (4) GEF-NPs (equal to 20 mg/kg free GEF). These compounds were administered every three days via intravenous tail injection, for a total of four times treatments. Drug dosage was based on initial body weight and not adjusted with weight change during the study period (Hsu et al., 2016). Weight and tumor volume were measured every other day. Tumor volume was calculated by the following formula:

$$volume=\frac{1}{2}\times length\times width^{2}.$$

To further study the antitumor effect against lung cancer, survival period, signs of reduced physical activity and tumor progression were evaluated. Six mice in each group were sacrificed by cervical dislocation at the end of the treatment period and tumor tissues were collected for immunohistochemical analysis, cell apoptosis assay and cell-cycle analysis.

### 2.9. Small-animal PET/CT imaging

In order to assess GEF-NPs therapy response and identify residual tumor masses after treatment, small-animal PET/CT imaging scans (Siemens, Germany) were acquired in three mice each group after 48 h of the fourth therapy. Briefly, food and water were removed to start a fast period of at least 6 hours prior to scan. Next, mice were anesthetized by an intraperitoneal injection of 1% pentobarbital 5 mL/kg, after at least 30 min from an intravenous injection of 150–250 μCi FDG 0.1–0.2 mL. PET/CT examinations were obtained in 2 D mode from the whole body (10 min of emission scan per bed position), while the other scanning parameters were 80 kV, 500 μA, and 1.5 mm slice collimation. Changes in [18 F] FDG uptake were compared among groups. The obtained PET/CT images were analyzed by two experienced nuclear medicine physicians. The regions of interest (ROIs) were manually drawn over the tumor to obtain the maximum standardized uptake value (SUVmax), which was calculated using the single hottest pixel inside the tumor.

### 2.10. Tumor apoptosis assay and cell-cycle analysis

Six mice in each group were randomly sacrificed by cervical dislocation. About 5 × 5 cm tumor mass was collected and used for apoptosis assay and cell-cycle analysis by flow cytometry (BD FACSVerse, San Diego, CA). Briefly, the prepared tumor tissue was cut into pieces and incubated in 1 mL trypsinization buffer for approximately 40 min at 37 °C. Blending was performed once every 5 min, and digestion was terminated after 40 min with the addition of medium containing serum. The tissue mixture was washed twice with PBS (pH = 7.4) and centrifuged at 1000 rpm for 2 min. Cells were harvested and filtered by a 70-μm nylon membrane to remove undigested tissue. Filtered cells were resuspended in Annexin-V binding buffer and incubated for 15 min with 5 μL AnnexinV-FITC and 5 μL PI, and apoptosis was evaluated by flow cytometry (BD FACSVerse) in 1 h. Cell-cycle assay was performed using BD cycle test plus DNA kit and also analyzed by flow cytometry.

### 2.11. Side effects evaluation and histopathological analysis

At the end of the experiment, heart, lung, spleen, liver, kidney and skin were collected to assess histological changes using hematoxylin and eosin staining and evaluated under microscope (Biological microscope, BX53, Olympus Corp, Japan).

To evaluate pulmonary and gastrointestinal toxicity, blood was collected from the eyeball after treatment and centrifuged at 10,000 rpm for 10 min at 4 °C, the serum was collected and stored at −80 °C for serum TGF-β1 and IL-6 measurement. TGF-β1 and IL-6 concentrations were analyzed using commercially available ELISA kits. At the end of the treatment period, approximately 50 mg pulmonary tissue was collected from each mouse and stored in a −80 °C refrigerator, and then, the concentration of hydroxyproline (HYP) was measured by hydroxyproline assay kit.

### 2.12. Ki67, CD31 and EGFR expression by immunohistochemical analysis

Tissue sections for immunostaining were obtained from formalin-fixed and paraffin-embedded tumor samples by Envision method. Firstly, paraffin section was conventionally deparaffinized using a graded series of xylene and ethanol solutions and washed with PBS. Secondly, after the antigen retrieval process in EDTA-repairing liquid and citrate buffer, sections were immersed in methanol H_2_O_2_ to remove endogenous peroxidase activity. Thirdly, slides were stained with anti-human Ki-67, CD31 and EGFR monoclonal primary antibodies and a biotinylated anti-mouse secondary antibody, followed by color development using DAB (3,3-diaminobenzidine), and hematoxylin solution was used for counterstaining. Finally, the finished sections were observed under microscopy.

Five ×400 fields from each tumor sample were used and the number of CD31-positive microvessels was expressed as the average of five fields. Ki-67 and EGFR labeling index was calculated in five randomly selected areas in each tumor sample, as the number of Ki-67 and EGFR positive cells/total counted at 400 × magnification.

### 2.13. Statistical analysis

Quantitative data were expressed as mean value ± standard deviation, and statistical analysis was performed using GraphPad prism version 5.0 (GraphPad Software, San Diego, CA). Comparisons of mean value were performed by Student’s *t*-test and one-way analysis of variance (ANOVA). Kaplan–Meier method was used to evaluate the survival time, while the log-rank test was used to compare two survival curves. *p* value of less than .05 was considered statistically significant.

## 3. Results

### 3.1. Nanoparticles physicochemical characterization

**Figure 1.**
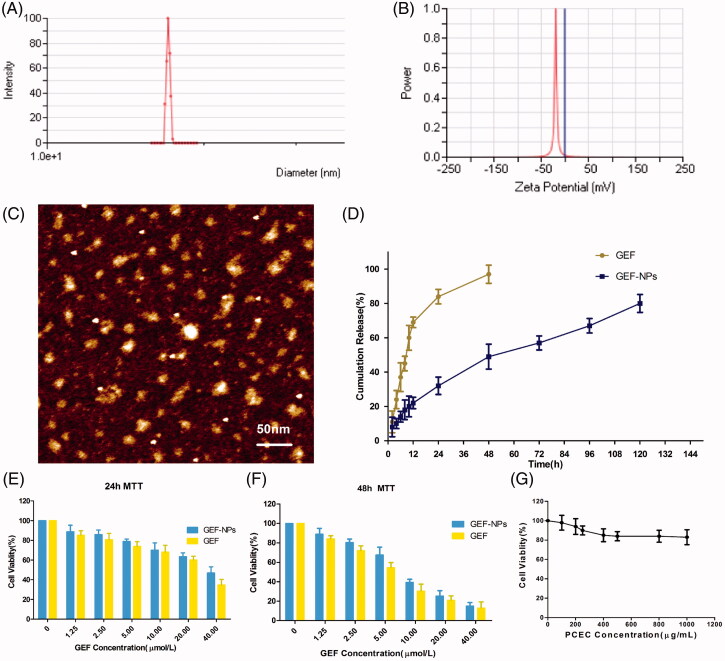
Physicochemical characterization of nanoparticles (A–D) and cytotoxicity study in vitro (E–G) (A): GEF-NPs particle size distribution; (B): Zeta Potential of GEF-NPs; (C): AFM image of GEF-NPs (scale bar =50 nm); (D): *In vitro* drug release of GEF-NPs and free GEF. Data are shown as means ± SD (*n* = 3). Cytotoxicity study *in vitro*: 24-h MTT and 48-h MTT was shown in Figure E and F, respectively. (G): The toxicity of PCEC to A549.

GEF-NPs were successful prepared by a solid dispersion method (The theoretical DL into GEF-NPs was set at 5%, 10% and 15% wt/wt). Nanoparticles with smooth surface and spherical shape could be clearly observed by AFM (Figure 1(C)), also showing well-dispersed character into an aqueous solution. They also had a narrow size distribution with an average diameter of less than 24 nm, while the polydispersity index (PDI) was less than 0.22 (Table 1). GEF-NPs zeta potential ranged from −13 mV to −22 mV and the results are shown in Table 1 and Figure 1. Zeta potentials falling between ±20 mV are desirable and infer electrical stability, while small zeta potentials may result in aggregated NPs and unsteadiness (Soria et al., 2008).

**Table 1.** Characteristics of GEF-NPs.

GEF/PCEC Mass ratio in feed	Size (nm)	PDI	Zeta Potential (mV)	DL (%)	EE (%)	Redissolve
5%	16.5 ± 0.4	0.14 ± 0.01	−14.9 ± 1.3	4.3	85.4	Easy
10%	17.2 ± 0.2	0.15 ± 0.01	−21.8 ± 0.9	9.2	92.2	Easy
15%	21.7 ± 1.0	0.23 ± 0.02	−18.2 ± 1.5	12.6	83.8	Difficult

According to Table 1, our result showed that with the increase in theoretical DL, particle size, PDI and the actual DL increased gradually: 5% showed the smallest particle size (16.45 ± 0.38 nm) and PDI (0.14 ± 0.01), and easy to redissolve to a clear solution; 10% not only showed a relatively smaller particle size (17.23 ± 0.19 nm) and PDI (0.15 ± 0.01), but also showed the highest EE (92.15 ± 1.63%), and the same tendency to redissolve in water; finally, 15% was difficult to redissolve into a clear solution that was actually the most important problem, since this characteristic did not meet the requirement for an intravenous injection. Thus, GEF-NPs 10% DL was considered in our further experiments.

### 3.2. *In vitro* release behavior

As shown in Figure 1(D), approximately 80% GEF was slowly released from GEF-NPs in a controlled and sustained behavior within 5 days, with no burst effect. However, free GEF exhibited a rapid release behavior, and more than 95% GEF was released into the medium within 2 days.

### 3.3. MTT assay

**Figure 2.**
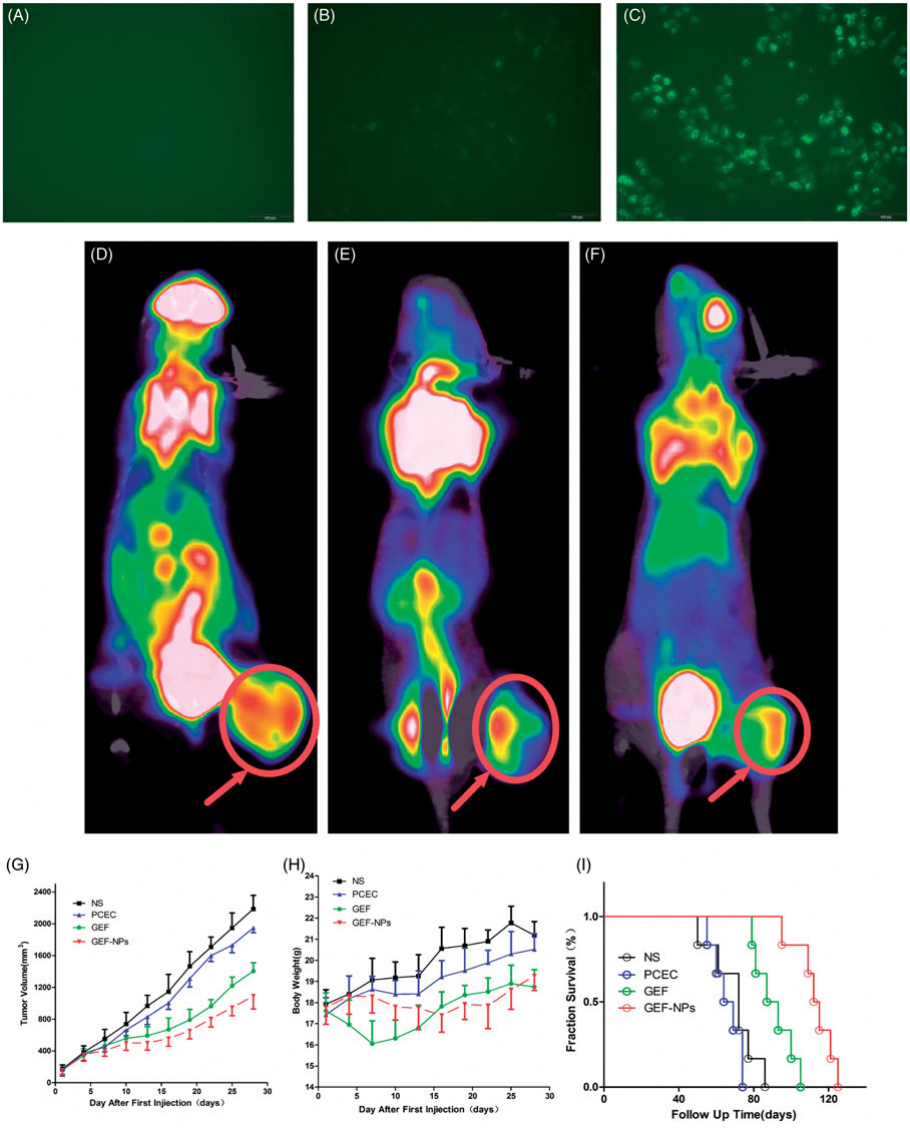
*In vitro* cellular uptake of PCEC polymeric micelles (A–C), example of each group mice on PET imaging (D–F) and evaluation of antitumor efficiency(G–I) A: blank control; B: The mixture of Coumarin-6 and PCEC; C:Coumarin-6-labeled PCEC nanoparticles (×400); (D):NS group, (E):GEF group, (F):GEF-NPs group; Evaluation of antitumor efficiency *in vivo* (G) and body weight changes (H) after treatment on A549 tumor-bearing nude BALB/c mice. Each point represents the mean of tumor size ± SD (6≤*N* ≤ 12). (I): The fraction survival of each group.

To inspect whether the released GEF was still pharmacologically active and evaluate GEF-NPs anti-tumor activity *in vitro*, MTT assay was performed on A549 cells. Figure 1(E–F) illustrates A549 cells viability after 24 and 48 h incubation with free GEF and GEF-NPs at different GEF concentrations (ranging from 1.25 to 40 μmol/mL). Cytotoxicity results indicated that cell growth inhibition induced by free GEF and GEF-NPs were dose and time dependent. A549 cells treated with GEF-NPs showed a higher viability than those treated with free GEF, as shown in Figure 2(E–F). This result might be due to the slow GEF release rate from the nanoparticles. The IC50 values for free GEF were 29.03 and 5.6 μg/mL after 24- and 48-h incubation, respectively, while the IC50 values for GEF-NPs were 37.8 and 8.1 μg/mL. This results indicated that GEF nanoparticles formulation was less cytotoxic compared with free GEF in the cell culture system. Figure 1(G) indicated that blank PCEC nanoparticles did not exhibit clear cytotoxicity to the cells with the increase of PCEC concentrations.

### 3.4. *In vitro* cell uptake

As shown in Figure 2(A–C), C6-labeled PCEC nanoparticles produced a stronger fluorescence than the other two groups after 2h incubation, indicating that C6-labeled PCEC nanoparticles could be taken up effectively by A549 cells.

### 3.5. Small-animal PET/CT imaging

A remarkable difference in tumor uptake values among the four groups was observed. The mean tumor SUVmax of the NS group was 3.1 ± 0.52 and varied significantly with a mean of 0.34 ± 0.12 for in the GEF-NPs group (*p* < .01 vs. NS group) and a mean of 1.10 ± 0.36 for GEF group (*p* < .05 vs. NS group). Example of each group mice on PET imaging was shown in Figure 2(D–F). Example of each group mice PET imaging is shown in Figure 2(D–F). Radioactivity was clearly lower in the tumors of the GEF-NPs group than those in the control groups, especially NS group.

### 3.6. Evaluation of antitumor effect *in vivo*

The groups treated with GEF-NPs and free GEF exhibited a significant tumor growth inhibition in comparison with NS and PCEC group (*p* < .01), as shown by the tumor growth curves in Figure 2(G). The tumor volume in nude mice treated with GEF-NPs increased slowly after treatment compared with the other three groups and continued the same tendency until the end of the experiment. After the first treatment of 28 days, GEF-NPs exerted a significant antitumor activity compared with the effect of the other treatments in the other three groups (*p* < .05 vs. all the other groups), Figure 2(G) also indicates that tumor growth was not affected by the treatment with blank PCEC nanoparticles compared with saline-treated tumors.

The body weight was measured every other day. When the first treatment began, no significant difference emerged in body weight between groups. A continuous weight loss occurred with the group treated with free GEF within 15 days, probably due to GEF administration into the blood stream causing damage to healthy tissue and consequent side effects. In contrast, the other three groups exhibited steady weight increase. Figure 2(H) indicates that GEF-NPs were efficient in preventing body weight loss compared with free GEF.

Survivals time are depicted in Figure 2(I). No evidence was found of a statistically significant difference in survival between NS group and PCEC group (*n* = 6), while Figure 2(I) also indicated that GEF-NPs (median survival time =113.5 days) had an better antitumor activity in nude mice models *in vivo*, including increasing quality of life and prolonging survival time, compared with GEF group (median survival time = 90 days, *p* < .01).

### 3.7. Cell apoptosis assay and cell-cycle analysis

**Figure 3.**
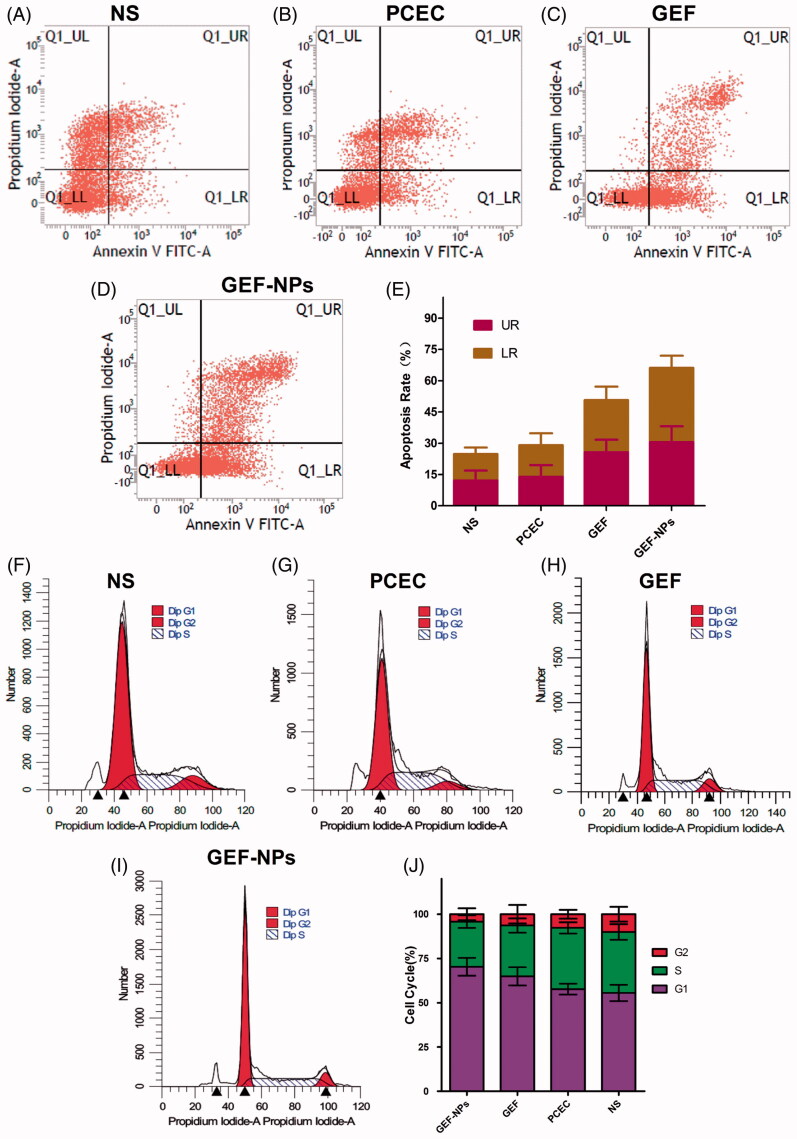
The tumor tissue apoptotic distribution (A–E) and cell-cycle distribution (F–J): (A–D): tumor tissue apoptotic distribution of different therapeutic effects on A549 tumor-bearing nude BALB/c mice; (E) Quantitative analysis of the proportion of cells in each group tumor tissue apoptotic distribution. (F–I): cell-cycle distribution of different therapeutic effects on A549 tumor-bearing nude BALB/c mice; (J): Quantitative analysis of the proportion of cells in each group cell-cycle distribution.

In order to confirm whether the cytotoxicity and antiproliferative effect induced by GEF-NPs treatment was due to apoptosis, flow cytometric analysis was performed. As shown in Figure 3, GEF-NPs group apoptotic rate increased from 50.69 ± 6.47% to 66.14 ± 5.84% compared with the GEF group (*p* < .05), while control group was 24.78 ± 3.17%. In addition, NS group and PCEC group necrotic cells rate was approximately 15% (Figure 3(A–B)), this result might be due to the rapid tumor growth without a rapid angiogenesis, suggesting no nutritional support for tumor cells especially in the center of the tumor that showed necrosis. However, GEF group and GEF-NPs group showed a higher percentage in late apoptosis rate (Figure 3(C–E)).

To gain further insight into GEF-NPs growth inhibition mechanism, we assessed cell cycle distribution by flow cytometry. As shown in Figure 3, G0/G1 phase in the GEF-NPs group was significantly increased from 55.5% to 70.33% compared with GEF group (*p <* .05), while G2/M phase was significantly decreased from 10.05% to 4.22% (*p <* .05), and also, the S phase was significantly decreased from 34.62% to 25.45% (*p <* .05). In addition, the sub-G1 peak, indicative of apoptotic cell death, was detected in the GEF group and GEF-NPs group. These results might indicate that GEF-induced apoptotic cell death and were also consistent with the apoptosis assay results.

### 3.8. Side effects evaluation and histopathological analysis

**Figure 4.**
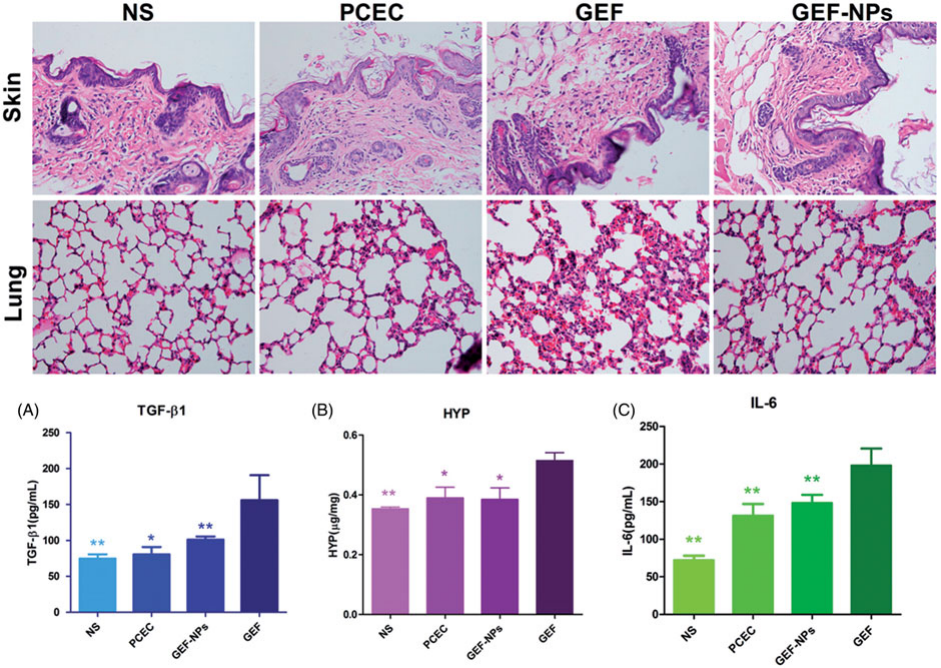
Side effects evaluation: H&E staining sections of skin and lung of each group (Original magnification, ×200): (A): The level of TGF-β1 in blood; (B): The content of HYP in lung tissue. (C): The level of IL-6 in blood. *p < .05 and **p < .01.

EGFR activation is associated with fibroproliferative processes in human lung disease and animal models of pulmonary fibrosis. To investigate the side effect of GEF on the lungs, we collected lung tissue and blood to analyze collagen fibers content. TGF-β1 level in blood plays a critical role in pulmonary fibrosis induced by GEF (Li et al., 2015), and TGF-β1 overexpression results in persisting pulmonary fibrosis (Warburton et al., 2012). Figure 4(A) shows that TGF-β1 level in the GEF group was 155.99 ± 34.87 pg/mL, while in the NS group was 74.94 ± 5.87 pg/mL (*p* < .01), in the PCEC group was 80.64 ± 10.35 pg/mL (*p <* .01) and in the GEF-NPs group was 101.20 ± 4.33 pg/mL (*p* < .05). All the *p* values were obtained in comparison with the GEF group. Figure 4(B) also shows that the GEF group had higher HYP levels (0.515 ± 0.026 μg/mg, *p* < .05, vs. GEF group), while the other three groups showed not statistically significant difference. All these results indicated that GEF-NPs could reduce the damage in the lung compared with GEF. Along with the rise of HYP and TGF-β1, histopathology of lung tissue in GEF group was also showing chronic inflammation and interstitial thickening after treatment, as shown in Figure 4.

No atypical papulopustular eruption emerged during GEF treatment. Histopathology of the skin of all mice also did not show any epidermal or dermal damage. Histopathologically, a T-cell infiltrate around the follicular infundibulum was not observed, which is associated with a suppurative folliculitis as shown in Figure 4 (hematoxylin and eosin; original magnification ×400). Probably, the cumulative dose did not reach the amount necessary to damage the skin. Furthermore, we did not observe any crissum inflammation or diarrhea in any mice, but IL-6 level in the blood showed clear differences in the four groups. As shown in Figure 4(C), GEF group IL-6 level was 197.82 ± 22.70 pg/mL, while in NS group was 71.72 ± 6.33 pg/mL (*p* < .01), in PCEC group was 131.16 ± 15.64 pg/mL (*p* < .01) and in GEF-NPs group was 148.05 ± 10.84 pg/mL (*p*  < .01). H&E stained sections of liver, spleen, kidney and heart of each group were observed under light microscope. No organ hemorrhage was found and there was no difference between groups. The results are shown in detail in the supplementary materials.

### 3.9 Immunohistochemistry

**Figure 5.**
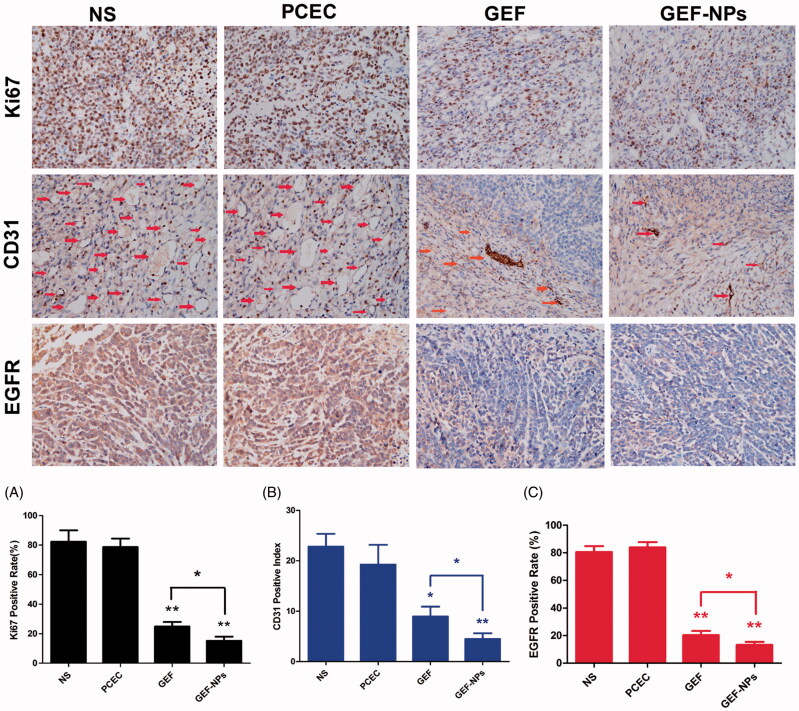
Representative images of immunohistochemistry analysis of each group for the evaluation of ki67, CD31 and EGFR. (Original magnification, ×400) The quantification immunohistochemistry analysis of each group for the evaluation of ki67, CD31 and EGFR were Figure A–C respectively. *p < .05 and **p < .01.

As shown in Figure 5(A): Ki-67-positive cells percentage was 82.62 ± 7.6% and 78.7 ± 5.8% in the NS group and PCEC group respectively, while GEF (24.9 ± 3.1%) and GEF-NPs (15.2 ± 2.8%) treatment induced a significant decrease of tumor proliferation compared with NS group (*p* < .01), with GEF-NPs showing better outcomes compared with the GEF group (*p* < .05), consistent with the tumor growth curve.

To evaluate GEF-NPs antiangiogenic potential, tumor tissue samples were routinely processed and immunohistochemically stained for CD31. The results (Figure 5(B)) showed that GEF-NPs group (4.50 ± 1.12) had lower vascular density compared with NS group (22.81 ± 2.67, *p* < .01) PCEC group (19.26 ± 4.01, *p* < .05) and GEF group (8.96 ± 1.94, *p* < .05). EGFR expression is also shown in Figure 5(C). GEF-NPs group had the lowest EGFR expression (13.2 ± 2.27%), while in the GEF groups was 20.27 ± 3.09% (*p* < .05), in the PCEC group was 80.5 ± 4.35% (*p* < .01) and in the NS group was 83.8 ± 3.92% (*p* < .01). All the *p* values were obtained in comparison with the GEF-NPs group.

## 4. Discussion

Lung cancer treatment is a challenging difficult problem with the increase in morbidity and mortality worldwide. Treatment outcomes and prognosis differ sharply because of lung cancer heterogeneity, and its evolution is unique in each patient in terms of histological and molecular feature (Burgess, 2011). Along with the breakthroughs in novel molecular-targeted therapy such as the one involving GEF, the greatest changes are mostly involving NSCLC treatment.

GEF is a selective EGFR-TKI and was the first approved for clinical use as an orally administered drug for patients with lung cancer. It was initially developed because improper activation of EGFR signaling appears frequently in lung cancer, thus, ASCO guidelines recommended GEF for the treatment of advanced NSCLC in second- or third-line settings (Azzoli et al., 2010). However, with the increasing use of GEF in clinical application, some problems came out, such as strong hydrophobicity, poor bioavailability and a wide range of tissue toxicity. The good news is that, in recent years, nanodrug system has emerged as a promising tool to improve bioavailability of poor water-soluble drugs. Therefore, it is interesting to develop a good formulation for the lipophilic GEF.

Nano-controlled GEF release system with hydroxypropyl β-cyclodextrin, chitosan, colloidal gold, liposomal and PLGA were already used for the design of formulations and showed enhanced antitumor effect *in vitro*, but all of them lacked evaluation of *in vivo* antitumor effect of GEF nanodrugs. In our previous study, we found that PCEC nanoparticles were potentially carriers for hydrophobic drug delivery due to the introduction of hydrophilic PEG segments into PCL backbones. According to our knowledge, no literature is available on PCEC copolymer used as GEF nanocarrier. Therefore, in this work, we chose the triblock PCEC polyether ester as a polymer matrix to fabricate a novel GEF delivery system, and we especially focused on the evaluation of GEF-NPs antitumor effect and side effects *in vivo*.

In this work, we successfully prepared GEF-loaded PCEC nanoparticles by a solid dispersion method without using any unwanted surfactants and vigorous stirring. These drug-loaded nanoparticles prepared by solid dispersion method have always small size and high DL compared with other methods as reported by recent articles (Chokshi et al., 2007). The obtained GEF-NPs were mono-dispersed (PDI  < 0.22), smaller than 24 nm and could be well redissolve in water to meet the requirement of intravenous injection. Many recent studies have highlighted the significance of nanoparticle sizes, which would greatly affect the fate of nanoparticle *in vivo*: less than 200 nm showed longer blood circulation time, greater stability, lower cytotoxicity and favorable uptake by EPR effect (Arshad et al., 2015; Muntimadugu et al., 2016). Moreover, the DL capacity and entrapment efficiency were in the range of 9.1–9.3% and 90–94%, respectively. The prepared PCEC nanoparticles showed no cytotoxicity on A549 cells *in vitro* and *in vivo* in nude mice, demonstrating that PCEC nanocarriers were safe as drug delivery system as many studies reported (Guo et al., 2011). GEF was released in a controlled manner from the nanoparticles, but *in vitro* might lead to a weaker antitumor effect because of the lack of cumulative amount of drug slowly released and EPR effect.

An inverse relationship between GEF slower release rate *in vitro* and higher cell toxicity was observed in MTT assay. A difference in cytotoxicity induced by GEF and GEF-NPs was observed after 24 and 48 h incubation. GEF-NPs exerted a lower cell inhibition ratio than free GEF. This might be ascribed to GEF slow release from nanoformulations. A longer incubation time may be necessary to evaluate the lack of significance in the toxicity results (Natarajan et al., 2014).

Then, we applied this novel GEF-NPs in a mouse model to discuss its antitumor effects and mechanism *in vivo*. GEF-NPs exhibited higher antitumor activity, small body weight changes and lower side effects compared with free GEF *in vivo* at equivalent doses. There are several explanations for the enhanced efficacy and relatively lower toxicity of GEF-NPs after intravenous injection. First, the improvement of GEF pharmacokinetics and prolonged circulation time resulted in higher accumulation in tumors due to the EPR effects (Fang et al., 2014), that is a passive targeting. Second, because GEF-NPs have a small size, they could easily accumulate in tumor tissue. GEF is released in a sustained manner, so that tumor cells can be exposed to GEF for longer time periods. Third, in patients whose tumors harbor EGFR mutations, GEF, as a EGFR-targeted agents, is superior to many other treatments in terms of response rates (Maemondo et al., 2010).

GEF-NPs led to a significantly longer delay on tumor growth and an increased median survival time of approximately 23 days compared with GEF group, and throughout the course of the GEF-NPs treatment, we did not observe a remarkable weight loss, while a continuous weight loss occurred in the group treated with free GEF within 15 days. As supported by side effects studies *in vivo*, GEF-NPs could minimize adverse effects compared with the GEF group, the content of TGF-β1 and HYP decreased 35% and 23%, respectively, along with higher HYP level and TGF-β1. GEF group H&E staining sections also showed chronic inflammation and interstitial thickening in lung tissue. In the whole treatment course, we did not observe crissum inflammation or diarrhea in any mice, but IL-6 levels in the GEF-NPs group had a decrease of 25% compared with GEF group. An atypical papulopustular eruption induced by GEF treatment was not found in skin of all mice, confirmed by the skin tissue histopathology, not showing any epidermal or dermal damage. It is important to note that in this study GEF-NPs had the potential to attain superior outcomes and to overcome complications associated with organ toxicities.

Many studies have confirmed that [18 F] FDG-PET is able to identify very early (i.e. only 24 h after treatment initiation) a decrease in glucose metabolism, which is correlated with overall survival and progression-free survival in many cancers (Ad et al., 2008). PET detection of new lesions early in the course of therapy has been reported to be a strong, independent predictive factor for overall survival in NSCLC patients treated with EGFR inhibitor (Aukema et al., 2010). In our study, small-animal PET/CT also confirmed that GEF-NPs has superior antitumor effect, as compared with free GEF or control group, GEF-NPs group had the lowest tumor uptake, which has a prognostic value in NSCLC (Hachemi et al., 2013). Furthermore, no new lesions detected by micro-PET early in the course of therapy were observed, in accordance with the result of survival analysis.

In order to further study GEF-NPs mechanism of action *in vivo*, the distribution of apoptosis and cell-cycle progression were analyzed using flow cytometry. The result suggested that GEF-NPs also induced cell-cycle arrest at G1-S boundary with apoptosis-inducing action (Shintani et al., 2004) as free GEF and showed the highest apoptotic rate in all groups. Tumor proliferative activity is usually assessed by Ki-67. CD31 is expressed on blood vascular endothelial cells and is considered as a marker of endothelial proliferation in patients with NSCLC (Suciu et al., 2015). Circulating microvascular density of the tumor has been established as prognostic factors in many studies (Nefedova et al., 2016). EGFR overexpression or activating mutation may promote tumor growth by increasing cell proliferation, motility, invasive capacity or by evading apoptosis, which were thus associated with poor prognosis (Lee, 2013). In our study, the GEF-NPs group showed the lowest expression of ki-67, CD-31 and EGFR after treatment compared with the other three groups. A low degree of cell proliferation, MVD and EGFR generally correlates with slow tumor growth, higher degree of differentiation and hence more moderate clinical and biological behavior (Bartoš et al., 2012).

Although GEF-NPs have the potential to attain superior outcomes and to overcome complications associated with organ toxicities, therapeutic resistance and disease relapse, scale-up production of GEF-NPs by solid dispersion technique has been a limitation in its developments to a formulation tool, and more detailed *in vivo* study on the GEF-NPs including its pharmacodynamics effects and in vivo release behavior, currently need further investigation. Also, the EGFR mutant NSCLC cell line will be used to verify the antitumor effect and its mechanism of GEF-NPs in our next study.

## 5. Conclusions

In this work, GEF-NPs were successful prepared by solid dispersion method and showed a strong time and dose dependent cytotoxicity against A549 cell line. In addition, GEF-NPs intravenous injection resulted in tumor growth inhibition, lower side effects and longer survival time in an *in vivo* animal model. Our results suggested that intravenous administration of GEF by PCEC nanoparticles might represent an alternative therapeutic approach for NSCLC treatment.

## Supplementary Material

IDRD_Fu_et_al_Supplemental_Content.doc
