# Supplementary material for: In vitro and in vivo antitumor effect of gefitinib nanoparticles on human lung cancer
Source: Drug Deliv. 2017 Sep 29;24(1):1501–12. doi: 10.1080/10717544.2017.1384862 (PMC8241075; doi:10.1080/10717544.2017.1384862)
Supplement: IDRD_Fu_et_al_Supplemental_Content.doc [file IDRD_A_1384862_SM5691.doc]

**Supplementary materials**

To evaluate side effects of GEF-NPs in normal tissue, H&E staining sections of liver, spleen, kidney, and heart of each group were observed under light microscope. No organ hemorrhage or metastatic tumor was found and there was no difference between groups. The results were shown in S1-2.


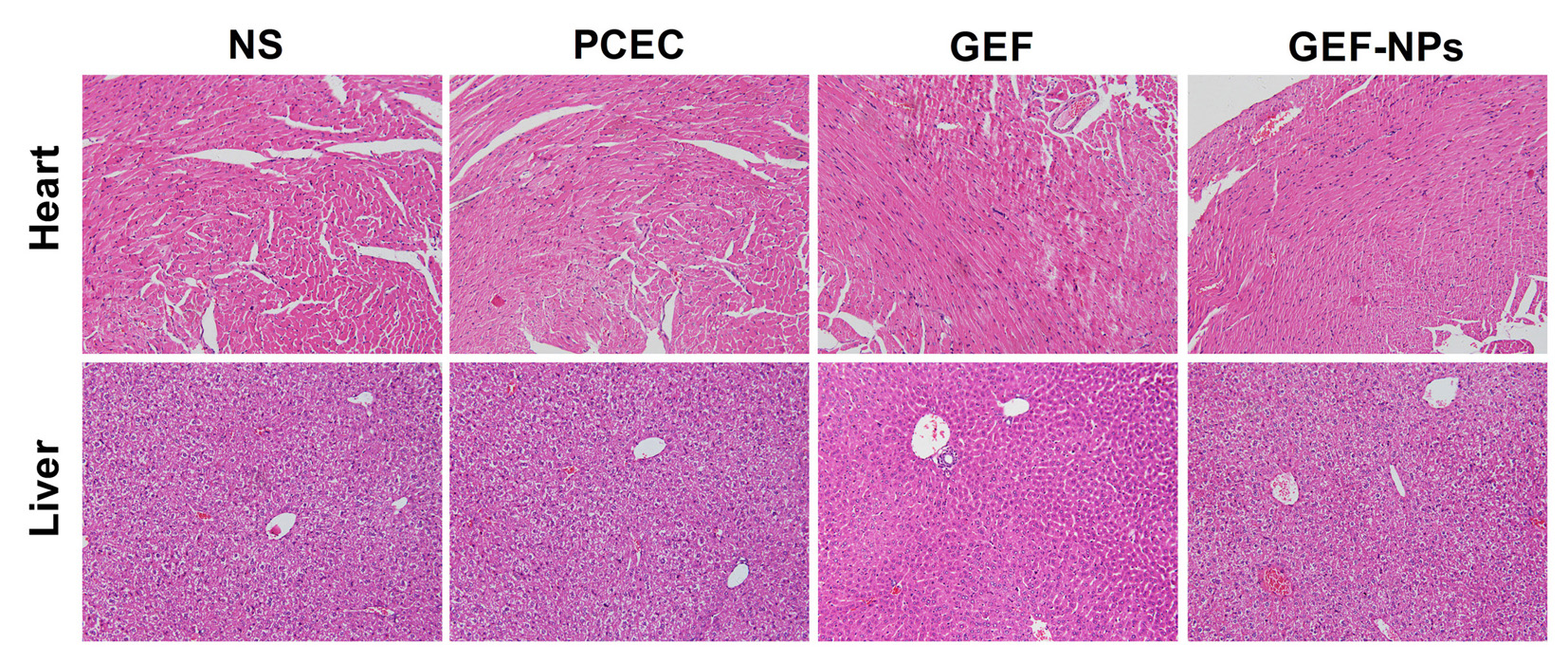


Figure S1 H&E staining sections of liver and heart of each group were observed under light microscope. (Original magnification, ×200)


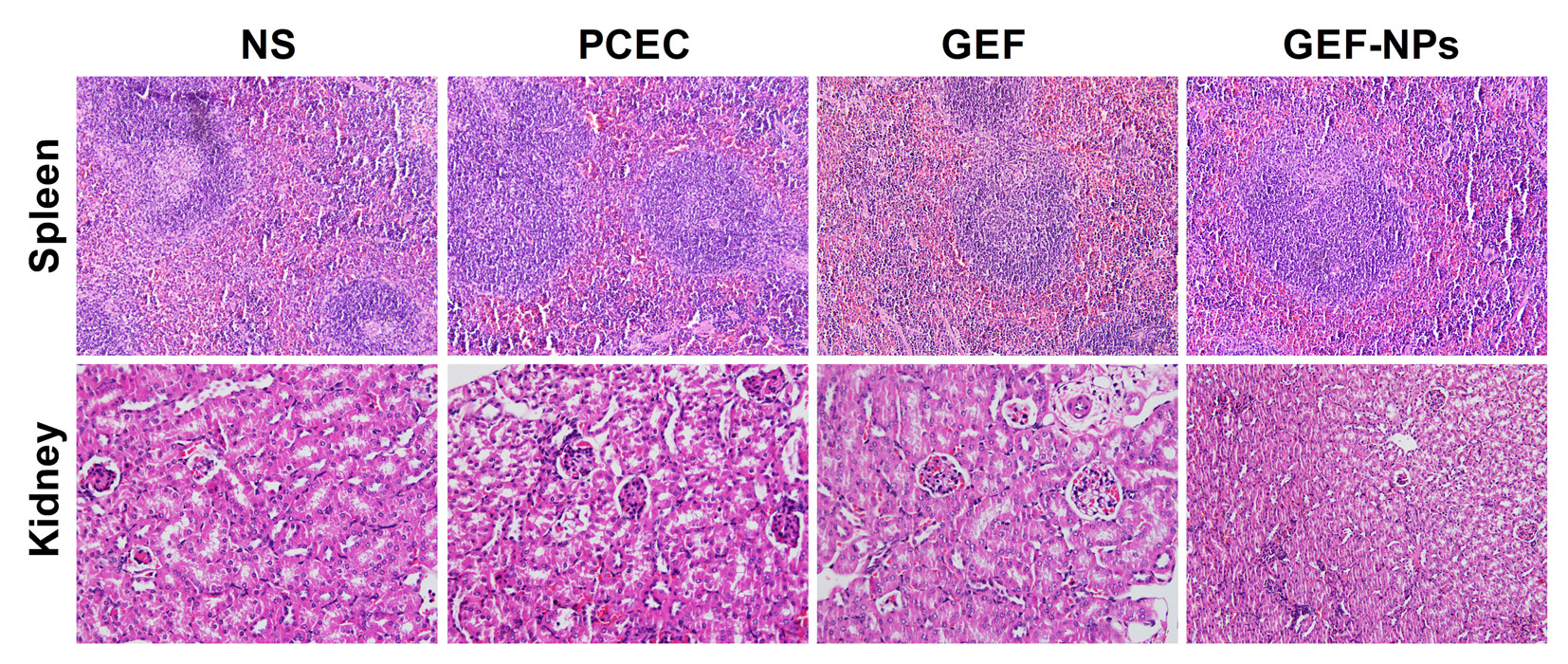


Figure S2 H&E staining sections of spleen and kidney of each group were observed under light microscope. (Original magnification, ×200)
